# RAVE: Comprehensive open-source software for reproducible analysis and visualization of intracranial EEG data

**DOI:** 10.1016/j.neuroimage.2020.117341

**Published:** 2020-09-10

**Authors:** John F. Magnotti, Zhengjia Wang, Michael S. Beauchamp

**Affiliations:** a Department of Neurosurgery, Baylor College of Medicine, United States; b Graduate Program in Statistics, Rice University, United States; c Department of Neurosurgery, Perelman School of Medicine at the University of Pennsylvania, United States

**Keywords:** Software, iEEG, Cortex, Analysis, Algorithms

## Abstract

Direct recording of neural activity from the human brain using implanted electrodes (iEEG, intracranial electroencephalography) is a fast-growing technique in human neuroscience. While the ability to record from the human brain with high spatial and temporal resolution has advanced our understanding, it generates staggering amounts of data: a single patient can be implanted with hundreds of electrodes, each sampled thousands of times a second for hours or days. The difficulty of exploring these vast datasets is the rate-limiting step in discovery. To overcome this obstacle, we created RAVE (“R Analysis and Visualization of iEEG”). All components of RAVE, including the underlying “R” language, are free and open source. User interactions occur through a web browser, making it transparent to the user whether the back-end data storage and computation are occurring locally, on a lab server, or in the cloud. Without writing a single line of computer code, users can create custom analyses, apply them to data from hundreds of iEEG electrodes, and instantly visualize the results on cortical surface models. Multiple types of plots are used to display analysis results, each of which can be downloaded as publication-ready graphics with a single click. RAVE consists of nearly 50,000 lines of code designed to prioritize an interactive user experience, reliability and reproducibility.

## 1. Introduction

The importance of high-quality software tools in advancing human neuroscience research is self-evident. The availability of purpose-built open-source fMRI software packages such as AFNI (Cox, 1996; Saad et al., 2006) and FSL (Smith et al., 2004) allowed thousands of neuroscientists and psychologists who were not experts in signal processing to analyze data from fMRI experiments. Similarly, while innovations in amplifier electronics slashed the cost of scalp encephalography (EEG), the development of comprehensive, freely-available analysis packages such as EEGLAB (Delorme and Makeig, 2004; Martinez-Cancino et al., 2020) played a key role in enabling EEG discoveries.

The last decade has seen an exponential increase in the numbers of studies investigating neuroscience questions using invasive recordings from the human brain (reviewed in Parvizi and Kastner, 2018). Recordings using grids of electrodes that sit on the cortical surface are referred to as electrocorticography (ECoG) while studies using depth electrodes that penetrate into the brain with recording contacts spaced at regular intervals along the shaft are referred to as sterotactic EEG (sEEG). Taken together, both recording techniques are referred to as intracranial EEG (iEEG). Because iEEG electrodes are implanted directly on or in the brain, iEEG recordings feature high spatial and temporal resolution, excellent signal-to-noise ratio and long continuous recordings without artifacts. Disadvantages of iEEG include sparse and variable electrode coverage, the presence of epileptogenic artifacts, and challenging experimental conditions in the epilepsy monitoring unit. As a result, iEEG datasets differ markedly from fMRI and EEG datasets. The development of RAVE was prompted by the limited options for neuroscientists in need of a comprehensive, open-source software package that handles all aspects of iEEG data analysis and visualization.

The design philosophy of RAVE is based on five principles. The first principle is *rigorous statistical methodology*. The history of fMRI has been marked by tumult over problematic statistics, such as “voodoo correlations” resulting from biased analyses (Simmons et al., 2007; Vul et al., 2009); treating subjects as fixed *vs*. random effects (Mumford and Nichols, 2009); and the “dead salmon” debate over how to correct for multiple comparisons (Bennett et al., 2009; Eklund et al., 2016; Cox et al., 2017). To encourage statistical best practices, RAVE is developed using “R”, a free, open source statistical language with a rich framework of existing packages developed by leading statistical and machine learning researchers (Computing, 2013). RAVE implements robust statistical tests, such as linear mixed-effects models, in a rigorous and community-vetted fashion (Bates et al., 2015; Kuznetsova et al., 2017).

The second principle is to *keep users close to the data* so that users may make discoveries about the brain without being misled by artifacts. This necessitates a well-designed graphical-user interface (GUI) so that users can explore very large iEEG datasets combined with an efficient, vetted algorithm implementation to display analysis results quickly enough for real-time, interactive interrogation.

The third principle is to *run anywhere*. RAVE is designed so that all user interactions can take place within a web browser. This makes RAVE platform and processor independent, running on tablets, desktops, or clusters. The RAVE front-end experience is the same whether data and computing resources are located on the user’s own machine, a lab server, or a cloud-based computing service such as Amazon Web Services.

The fourth principle is to prioritize *reliability and reproducibility* in all aspects of RAVE development. Funding agencies and journals have recognized that data sharing is a key ingredient in speeding scientific progress, with all research programs supported by the United States National Institutes of Health Brain Research through Advancing Innovative Neurotechnologies (BRAIN) required to submit their research data to an approved archive (Zhan, 2019). Archive submission often entails a laborious collection and curation process on the part of investigators. RAVE simplifies this process by automatically harmonizing all iEEG data files from each participant, together with meta-data such as task epoch files, in a format acceptable to archives. All processing commands used to generate a particular file or analysis can be easily documented for attachment to manuscript figures.

The final principle is to *play well with others*. Each laboratory has its own ecosystem of software tools and methods with expertise and protocols developed over years of experience. Therefore, RAVE is designed to integrate seamlessly with existing workflows, such as iELVis (Groppe et al., 2017) and img_pipe (Hamilton et al., 2017) for electrode localization. Along with an extensive GUI, RAVE provides a robust application programming interface (API) to support integration of RAVE with existing or novel analysis pipelines.

## 2. Methods and results

RAVE source code and documentation are available at https://openwetware.org/wiki/RAVE. Text and video tutorials allow users to quickly learn the essential elements of iEEG analysis with the included sample dataset. RAVE consists of interpreted R code and uses the R Shiny package (Chang et al., 2019) to create interactive web apps. The user’s interactions with these web apps via a web browser constitutes the RAVE graphical user interface (GUI). Each element of the GUI shows an information button (“?”) that links to a web page with helpful descriptions. RAVE has been tested on Mac, Windows, and Linux platforms. A Docker instance is available for easy distribution of RAVE with all dependencies and a demo dataset.

### 2.1. Deployment strategies

**Fig. 1.**
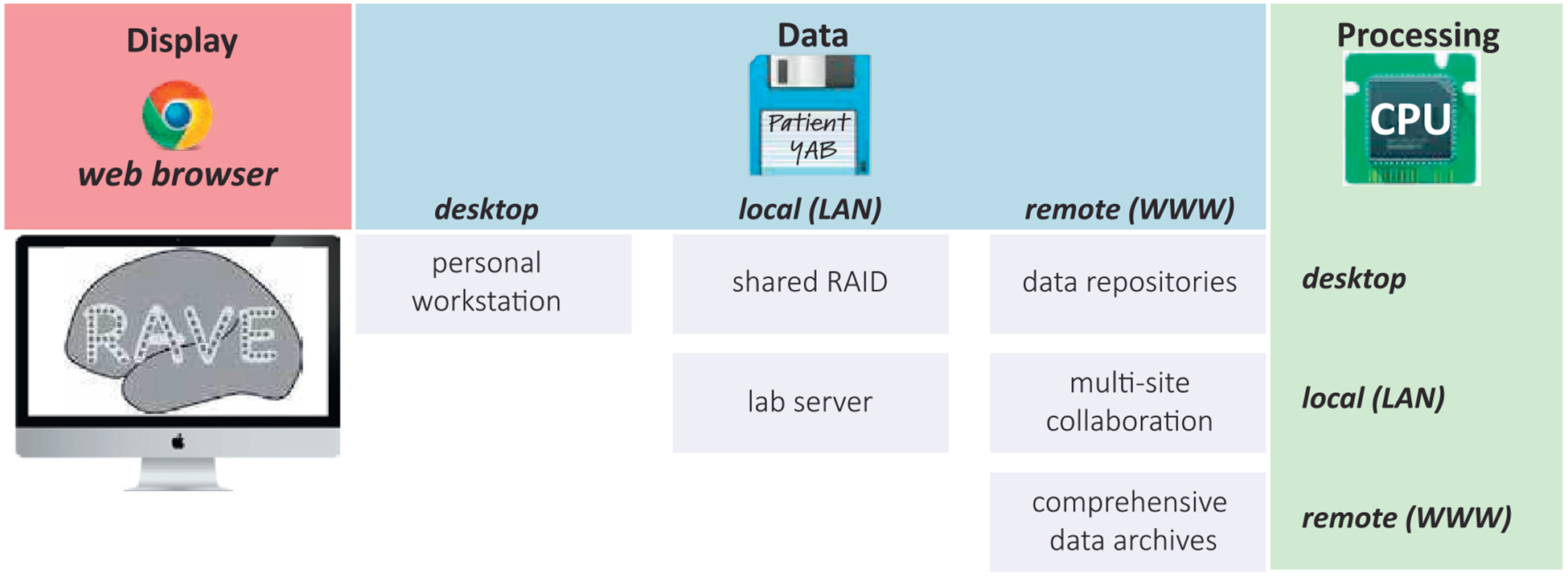
Schematic of RAVE deployment configurations. Users interact with RAVE via a web browser running on a desktop, laptop, or tablet (red “Display” column). Data storage (blue, columns) and processing (green, rows) can both occur on the same desktop machine used for display. This is mandatory in situations with little or no internet connectivity, such as a hospital room or an airline flight. In laboratory situations, processing can occur on the desktop while data storage is shared, or both processing and storage can be shared. For large-scale collaborations, data can be stored remotely (*e.g.*, using a cloud storage system) and processing can occur either on the desktop, locally, or in the cloud.

The user always interacts with the RAVE GUI using a web browser on a local machine (desktop, laptop or tablet). The location of the data repository and the hardware used for analysis computations may be independently configured, creating a variety of possible deployment strategies (Fig. 1).

In hospital environments, internet access is limited (an investigator preparing a manuscript on an airplane is similarly challenged). In these situations, RAVE can run on a local machine such as desktop or laptop with the user interactions taking place through a web browser on the same machine. Both data storage and computation take place on the local machine.

In laboratory environments, inexpensive shared data storage is often used so that lab members may access data from many different projects and subjects (a 20 TB RAID can be purchased for less than $1000USD). In this scenario, RAVE runs on each user’s local machine, controlled via web browser. Analyses occur locally, but data is stored on the shared RAID (typically accessed via a mount point).

A third type of deployment places analysis at a laboratory level on a shared lab compute server, such as a cluster. In this configuration, multiple RAVE sessions are run in parallel on the server, controlled by a web browser on each user’s local machine. Analysis and storage take place on the central compute server/RAID. This configuration has two advantages. First, compute servers can be equipped with dozens of cores and TB of RAM, increasing processing speed. Second, large data files are transferred exclusively over fast connections between the compute server and the RAID (both of which would often be hosted in a university data center) rather than slower desktop connections between the data center and the desktop. Network performance for desktop connections can vary, especially for telework situations in which the user is connecting via virtual private network (VPN).

Other deployments are also possible. An investigator might upload a large library of iEEG data to an online repository (Miller, 2019). Users running RAVE on their local machine could set the data location to the online repository, resulting in local analysis with remote storage. Alternately, in a multi-lab collaboration, a private data repository could hold all data collected across labs, with each lab’s compute server connecting to the repository for laboratory-level analysis with remote storage.

A final deployment strategy places both analysis and storage “in the cloud.” This would be appropriate for a comprehensive data repository, such as those under construction for the NIH BRAIN Initiative including NEMAR (Franklin, 2019) and DABI (Toga et al., 2019). A user would run a web browser on their local machine and point it to an instance of RAVE running on an academic or commercial cloud computing service, which would also host the entire data archive.

## 3. Software architecture

**Fig. 2.**
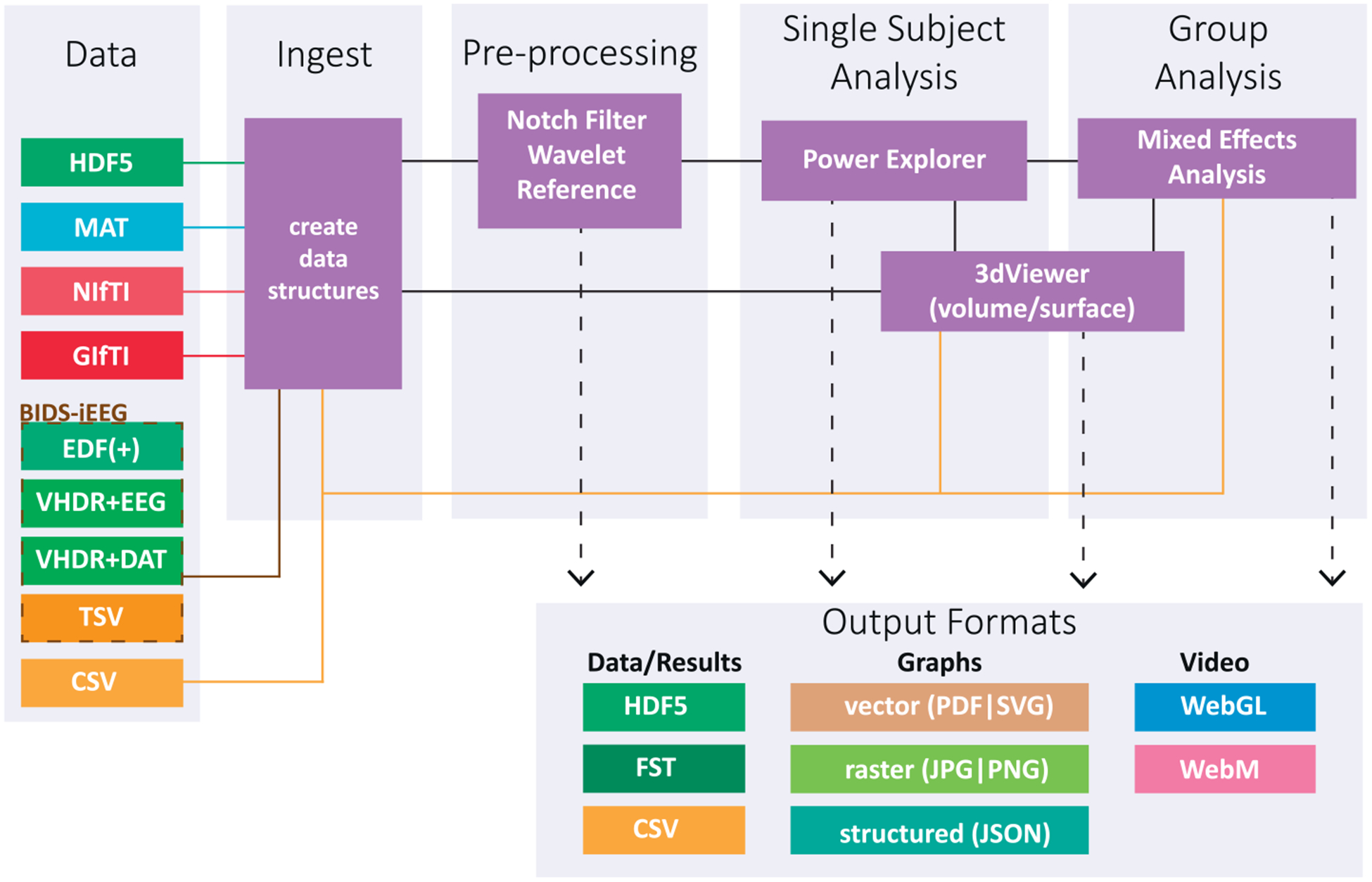
Flowchart of RAVE software architecture, proceeding from data input, through different processing stages, and ending in output of processed data and results, graphs and plots, and videos. Data files are stored internally using the HDF5 format and can be imported from a variety of formats. MRI volumes are imported in standard NIfTI format and cortical surface models in GIfTI format. The format for exported data varies depending on the type of data being exported.

Fig. 2 shows a flowchart of the components of the RAVE software most relevant to users. Using the GUI or command line calls, the user identifies the location of raw iEEG data files. For visualization, users can also identify the location of MRI images of the patient’s brain and cortical surface models created from the MRI images with FreeSurfer (Dale et al., 1999; Fischl et al., 1999a) or other reconstruction tools. If a subject MRI is not available, data can be displayed on a standard template brain.

A typical iEEG patient might perform many different tasks in the course of their hospitalization. For instance, in subject 1, some data files might be a speech task, others a vision task, and still others a memory task, while in subject 2, the same tasks might be run in a different order. RAVE reorganizes all data files into a consistent structure, using a directory tree with project (such as speech, vision or memory) at the highest level, followed by subject, followed by data files. Data files are stored in the HDF5 format, the same format used by Neurodata Without Borders (Teeters et al., 2015) and recent versions of Matlab. As detailed below, spectral decomposition is performed by the RAVE pre-processor, then data exploration in single subjects with the *Power Explorer* module and across subjects with the *Group Analysis* module. In *3dViewer*, users can click on individual electrodes and view their activity profile, localizing them on the cortical surface for ECoG electrodes or on a 3-plane view of the MRI volume for sEEG electrodes.

A variety of output options are available. High-quality PDFs or PNGs of any graph or brain image can be exported with a single click from each module for use in figures within presentations, manuscripts, and grant proposals. The data underlying any analysis can be exported in text or Microsoft Excel format with a single-click to create manuscript tables or for analysis outside of RAVE. Animations showing brain activity evolving over time can also be generated easily (in .*webm* format), or even exported as a standalone web app complete with all functional and anatomical data (HTML and JavaScript using WebGL).

To further the design principle of playing well with others, there are multiple entry and exit points for the processing stream: users are not locked into a sequential analysis that begins with raw iEEG data and ends with an activity plot. For instance, a clinician could visually inspect the clinical recordings from each electrode and assign an index of epileptiform activity to each electrode in a spreadsheet table. The clinician could then import this table into RAVE and display it on the cortical surface and 3-plane viewer, completely bypassing the preprocessing and analysis of the voltage-by-time data. Alternately, a data scientist could use the RAVE data structures and preprocessing modules to quickly and easily generate a single value for each condition in each trial in each electrode, then import this data into a machine learning toolbox for training and testing with leave-one-out analyses.

### 3.1. Single subject analysis

**Fig. 3.**
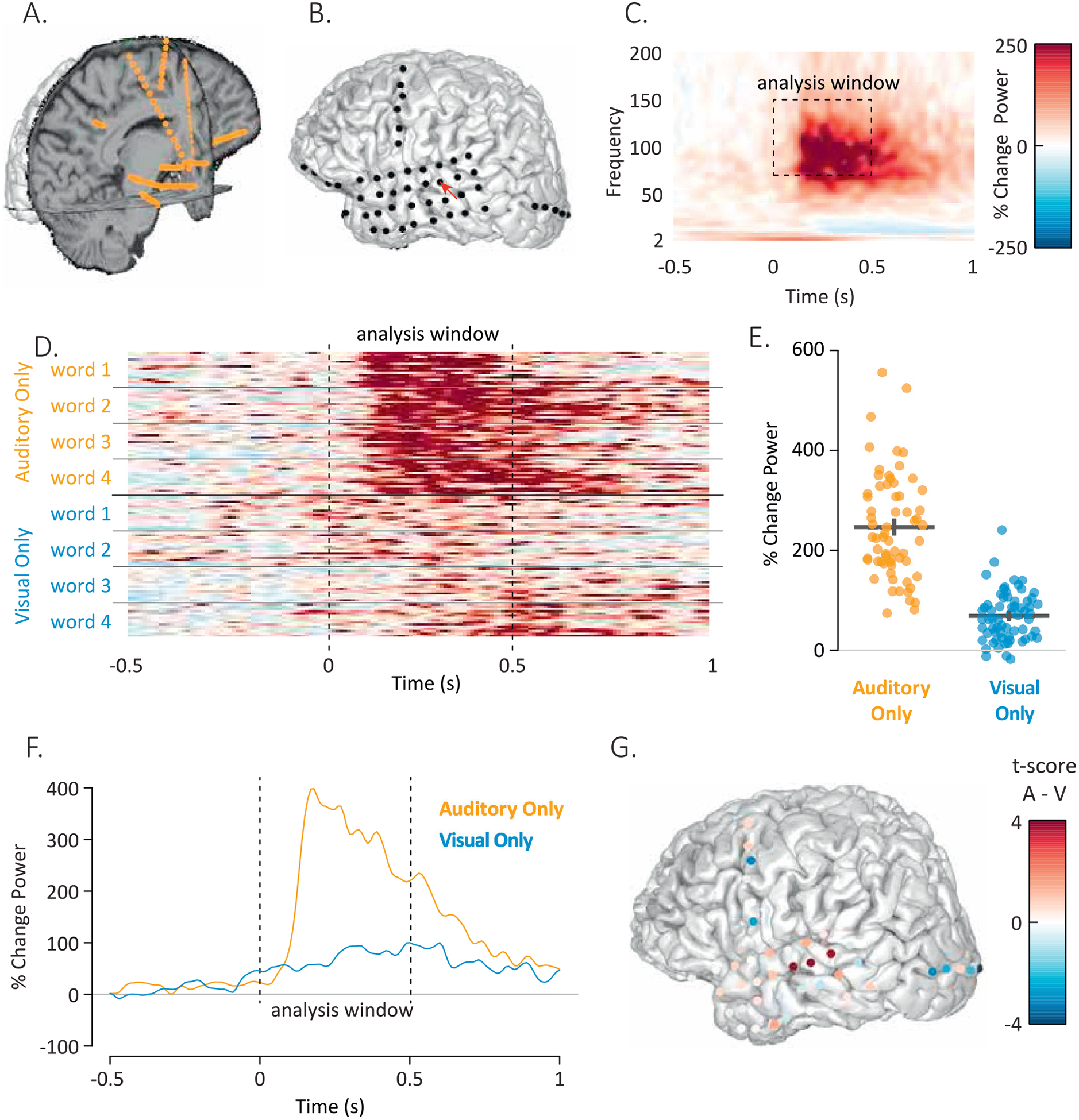
A. The *3dViewer* displays axial, sagittal and coronal slices through the MRI volume for localization of sEEG electrodes (orange spheres). B. The *3dViewer* also supports display of cortical surface models for localization of ECoG electrodes (black spheres). Red pointer highlights YAB electrode 14. C. Power-by-frequency plot showing the average response across words in YAB electrode 14. The experiment consisted of repeated presentations of single words, time zero corresponds to the onset of the auditory component of the word. D. Individual trial data from YAB electrode 14. Each row/strip shows the response in a single trial over time, collapsed across the frequencies within the analysis window. There were 8 total stimuli, 4 stimuli consisted of auditory-only recordings of words, 4 stimuli consisted of silent visual-only videos of the same words. There were 16 presentations of each individual stimulus. E. The response from each row/strip in (D) was converted to a single value by averaging over the analysis window from 0 seconds to 0.5 seconds. Each orange symbol shows the response to a single auditory-only trial, each blue symbol shows the response to a single visual-only trial. The black lines show the mean and standard error of the mean for each condition across trials. F. The average response to auditory-only words and visual-only words over time in YAB electrode 14, collapsed across the frequencies in the analysis window. G. The *t*-score of the power difference between auditory-only and visual-only conditions, calculated for each electrode (red: auditory only > visual only; blue: visual only > auditory only).

The heart of the RAVE user experience is fast and interactive data exploration. While signals from neighboring scalp electrodes in EEG (or sensors in MEG) are usually similar, neighboring electrodes in iEEG often respond completely differently as the electrode grid traverses functional boundaries in cortex (for ECoG) or the electrode shaft penetrates different subcortical nuclei (for sEEG). This makes accurate visualization, selection and display of individual electrodes a critical step in iEEG analysis. RAVE accomplishes this task with the hardware-accelerated *3dViewer* for electrode visualization and selection that can be embedded in a given module. ECoG electrodes are displayed on a cortical surface model of the participant’s cerebral hemispheres created by FreeSurfer or another reconstruction tool (Dale et al., 1999; Fischl et al., 1999a). The *3dViewer* cortical surface model view can be zoomed or rotated with the mouse or keyboard shortcuts Users can view the MRI volume in separate panels or overlaid on the cortical surface module, and then scroll through the slices (Fig. 3A). Individual electrodes are clickable, which then updates the current analysis to focus on that electrode (Fig. 3B).

*Power Explorer* displays several different kinds of plots to provide an in-depth view of a subject’s iEEG data. One set of statistical plots displays data for all electrodes in the subject, for a global view. The remaining plots display data for single electrodes or subsets of electrodes, selected either with the *3dViewer* or by setting anatomical or functional criteria.

The first type of plot is the time-by-frequency plot (Fig. 3C). The *x*-axis of the plot shows time from a given experimental event, such as the beginning of a trial or the onset of a stimulus. The *y*-axis shows frequency range with the power at each time-frequency cell mapped to a color scale. Both the color scale range, colors, and units of analysis (such as percent amplitude or power change from baseline, *z*-score of amplitude or power change from baseline, or dB from baseline) are user modifiable with a single click. For different analyses, the time-frequency range of interest differs. The range can be changed with sliders bars in the GUI, with the selected range shown as a dashed box on the spectrogram.

The iEEG signal-to-noise ratio is large enough that responses can be observed within individual trials. So that users can view the variability of the signal across individual trials, the GUI plots the response in the selected frequency range over time for each individual trial (Fig. 3D). The trials can be sorted by stimulus or experimental condition so that users may visually inspect the consistency of the signal and determine differences between stimuli or conditions.

The interface uses autofill text boxes so that users can group different trial types into an unlimited number of combinations (the default is to group all trials into a single condition). Analysis results are instantly updated to reflect any changes. Consider an experiment with 8 trial types where each trial consists of a recording of one of four single words presented in either auditory or audiovisual format. With a few clicks, the user could group all auditory words into an “auditory” condition and all audiovisual words into an “audiovisual” condition. Alternately, each trial type could be treated as an independent condition. The composition of the conditions can be saved to disk and reloaded to avoid having to recreate complex groupings for different subjects in the same project.

The activity for each defined condition is displayed in its own time-frequency plot. To display all conditions in a single plot, the data is collapsed across the time window of interest to generate a single value for each trial. Each trial is then plotted as a single point, with one column of points per condition (Fig. 3E). If the user has selected more than one condition, a statistical test is automatically performed between the conditions with the results displayed above the trial plot. To compare the temporal profile of the response across conditions, the spectral signal is collapsed across the frequency range of interest and displayed, with one trace of power over time per condition (Fig. 3F).

The results of the analysis across electrodes are visualized by coloring each electrode (Fig. 3G). A pull-down menu permits users to select the values used for coloring from all available values for single conditions and contrasts between conditions, including raw beta-weights, *t*-statistics, *p*-values, and false-discovery-rate (FDR) corrected *p*-values.

### 3.2. Group analysis

Discoveries made at the single subject level must be confirmed across subjects, requiring the creation of a common anatomical space. RAVE offers several choices. For electrodes located near the cerebral cortex, the most accurate approach is to use the surface-based coordinate system generated by FreeSurfer (Fischl et al., 1999b; Argall et al., 2006). For subcortical electrodes, RAVE uses the MNI-305 volumetric standard space generated by FreeSurfer; MNI-305 space can also be used for cortical electrodes if desired. A coarse-grained approach is provided by the FreeSurfer cortical and subcortical parcellation schemes (Fischl et al., 2004) in which electrodes are grouped by region-of-interest rather than co-ordinates.

The first step in the RAVE group analysis workflow is to identify the electrodes from each individual subject that are to be included in the group analysis, analogous to the voxel selection step in BOLD fMRI (a study of the fusiform face area might select voxels in each subject that are located in the fusiform gyrus and show a significant response to faces). In RAVE, up to three functional and two anatomical criteria can be combined to winnow down hundreds or thousands of iEEG electrodes into an appropriate subset. Anatomical labels from each electrode are taken from the electrode meta-data file, and functional criteria can be any of the statistical values generated by *Power Explorer* or other modules. For example, in a study of speech perception, electrodes in each subject might be selected using the anatomical criterion of “location on the superior temporal gyrus” and the functional criterion of “significant response to speech (*p* < 0.01, FDR corrected).”

**Fig. 4.**
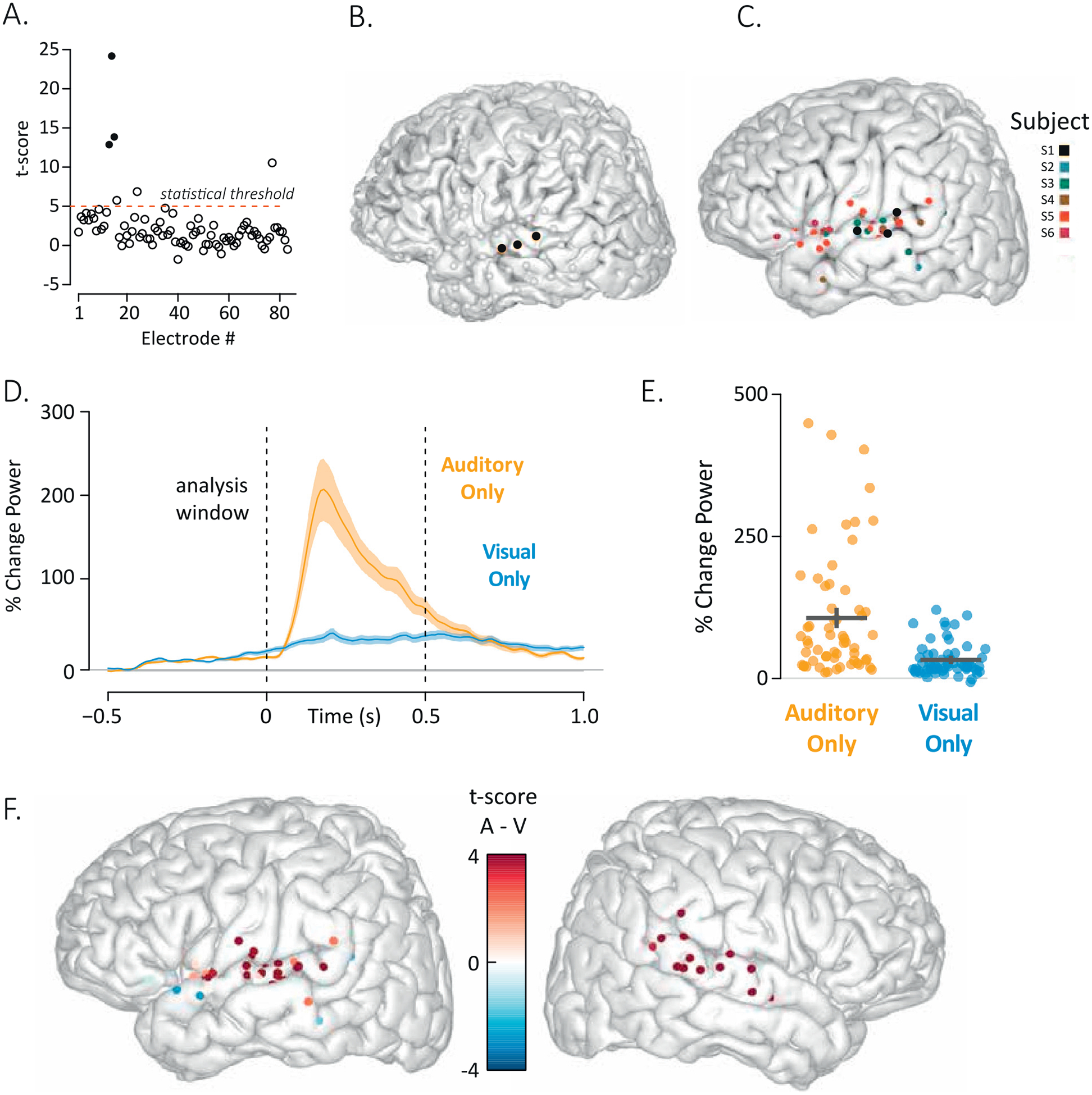
A. To visualize activity across all electrodes in a subject, RAVE plots a symbol for each electrode (*x*-axis is electrode number) with the *y*-axis displaying the result of the selected statistical test, in this case the *t*-test for the response to auditory-only words. Users may select electrodes for inclusion in the group analysis using combinations of up to three functional and two anatomical criteria. Here, the functional selection criterion is the statistical threshold (shown as a dashed red line) above which an electrode is considered responsive (*t* > 5 for this analysis). The anatomical selection criterion for this analysis was “located on the superior temporal gyrus”. Filled dots indicate electrodes that met both the functional and anatomical criteria, empty dots did not meet both criteria (empty dots above the red line indicate electrodes that passed the functional criterion but not the anatomical criterion). B. All electrodes in subject YAB displayed on the subject’s cortical surface model. Black electrodes met both anatomical and functional criteria in (A), gray electrodes did not. C. The same anatomical and functional criteria developed in (A) were applied to 832 electrodes across eight subjects. A total of 60 electrodes met both criteria. The locations of all left hemisphere electrodes that met the criteria are plotted on a template brain, colored by subject number (two subjects had only right hemisphere electrodes, not shown). D. The mean response across the 60 selected electrodes to auditory-only and visual-only words are plotted. Shaded regions indicate the standard error of the mean across electrodes. E. The response over time in each electrode was converted to a single value by averaging over the time window from 0 s to 0.5 s. Each orange symbol shows the response of a single electrode across all auditory-only trials, each blue symbol shows the response of a single electrode across all visual-only trials. The black lines show the mean and standard error of the mean for each condition across trials. F. Results of the statistical contrast between the auditory-only and visual-only conditions was calculated for each electrode and used to color the electrodes, displayed on the left and right hemispheres of a template brain.

An important analysis decision is the choice of the statistical threshold. To aid in this choice, RAVE displays a plot of the statistical value for each electrode along with a red dashed line showing the currently selected threshold (Fig. 4A). *3dViewer* can be updated to display only the selected electrodes (Fig. 4B). The entire power-by-time information for each individual trial for all selected electrodes is exported from *Power Explorer* with a single click. After this process is completed for each individual subject, the *Group Analysis* module is loaded with data from all subjects’ selected electrodes. The number and location of the electrode contributed by each subject can be visualized by coloring each electrode according to the source subject (Fig. 4C).

Because all information for all selected electrodes is loaded, the *Group Analysis* module lets users explore different analysis windows and condition contrasts for all electrodes in a study, in the same way as is possible for individual subjects in *Power Explorer.* Group contrasts and analysis windows are automatically prepopulated based on the single-subject analysis settings but can be changed.

Next, a linear mixed effect (LME) model is used to perform statistical tests across all electrodes and subjects. The default for the dependent variable is the unit of analysis (*e.g.* power change from baseline). The model defaults to treating electrode and subject as random effects, with electrode nested within subject in a multi-level fashion. Users can easily select which variables should serve as random or fixed effects, such as adding stimulus exemplar as a random effect (Westfall et al., 2016). The formula provided to the LME package is displayed, *e.g.* for a model without a fixed effect and the default random effect structure: Power ~ 1 + (1|Subject/Electrode).

The results of the group analysis are shown in a variety of tables and plots. The tabular output includes: random effect counts and variances; parameter estimates, standard errors, degrees of freedom and hypothesis tests for the fixed effects; the omnibus fixed effects test (equivalent to the main effect of condition); the comparison of all fixed effects against zero; and all pairwise comparisons between the fixed effects.

There is also a sortable and searchable table showing univariate statistical tests for each electrode included in the group analysis. Rows and columns from this table can be selected in the GUI and plotted. This is important for quick examinations of response differences between regions, subjects, and any other experimental variable.

The plots available in *Group Analysis* are similar to those available in *Power Explorer*, except they are created at the group level. The power-by-time plot shows one trace per condition, but estimates of variance are calculated across all electrodes included in the analysis to provide a graphical representation of the uncertainty in the mean (Fig. 4D). To examine the consistency of the effect across electrodes, the value in each condition for each electrode is plotted (Fig. 4E). An anatomical representation of the analysis results is created by coloring each electrode by the results of the selected statistical test (Fig. 4F).

A unique aspect of the *Group Analysis* module is the ability to create custom graphs. Using the “post-hoc plot” panel, the user can select “*x*” and “*y*” plot variables from a drop-down menu that contains all variables from the analysis output. A user might wish to assess whether an electrode’s response in condition 1 predicts the response in condition 2. Selecting the appropriate variables in the drop-down menu, the module creates a plot and performs the selected statistical test, including Pearson and Spearman correlations and *t*-test or Wilcoxon test of differences.

To assess the relationship between two variables while holding a third variable constant (partial regression), users can also select a third (“*z*”) variable. In an experiment with three conditions, a user might wish to assess whether an electrode’s response in condition 1 predicts the response in condition 2, partialling out the response in condition 3.

The panel also support creating variables from “R” code entered into a text box by the user; for example, to assess whether the *difference* in response between two conditions predicts the response in a third condition.

All plots and tables can be easily downloaded as high-resolution PDFs for manuscript or presentation figures, or as CSV files for use in manuscript tables or additional analyses.

### 3.3. Preprocessing and referencing

Because preprocessing of iEEG and EEG data are similar, the preprocessing workflow in RAVE is based on that in the widely-used EEGLAB package (Delorme and Makeig, 2004). To assess data quality, a variety of plots for each block of data and each channel are generated. RAVE creates a PDF showing data quality analytics, with one electrode per page, so that users may scan for problematic data. Analytics include plots of voltage *vs.* time after notch filtering; periodograms before and after notch filtering (both normal and log_10_ frequency); and histograms signal voltage. Spectral analysis is performed using wavelets with default parameters of 16 kernels with lengths from 0.101 to 1.433 seconds and cycles from 3 to 16 (Cohen, 2014). After waveletting, data are down-sampled to 100 Hz to reduce disk space; this value is also customizable. RAVE provides tools for semi-automated trial epoching based on signals present in the iEEG data, such as acquisition system analog inputs from microphones signaling the onset of auditory events or from photodiodes signaling the onset of visual events. Users can also provide a CSV file that contain the times of events within each trial, such as auditory onset, visual onset, or motor response. In *Power Explorer*, a drop-down menu lists all available trial reference timepoints and analyses are time-locked to the selected event.

A key step in EEG analysis is the choice of voltage reference. RAVE incorporates three popular reference schemes: common average reference, white matter reference, and bipolar referencing. Each of these schemes has advantages and disadvantages (Mercier et al., 2017; Li et al., 2018). With the common average reference, the average of the signal at all electrodes is subtracted from the signal at each individual electrode at every time point. In some clinical situations, there may be a preponderance of electrodes over a single brain area, such as cortex important for speech or motor functions. This concentration is strongly weighted in the common average, with the result that subtracting the common average removes neural signals of interest. In practice, experimenters are well-advised to analyze their data using different schemes to understand their influence on the results.

To make referencing faster and allow users to easily inspect how different referencing techniques influence results, RAVE flips the usual order of operations. In most EEG processing pipelines, data are referenced and then spectral analysis is performed. However, spectral analysis is by far the most time-consuming step of the analysis and expands data volume many-fold, making it tedious and inefficient to repeatedly select a different reference and re-run spectral analysis. Instead, RAVE performs spectral analysis first, followed by referencing. Because referencing is a purely arithmetic operation, it can be performed quickly, and users can quickly assess the effects of different reference schemes. Mathematically, referencing is a linear combination, and waveletting is a linear operator, with the result that the order of operations does not change the final result as long as both the real and complex components of the spectral signal are preserved.

## 4. Discussion

RAVE provides an easy-to-use software tool so that users with no programming or signal processing expertise can analyze iEEG data and create publication-ready figures and plots.

Direct recording of neural activity from the human brain using implanted electrodes is one of the fastest-growing techniques in neuroscience. Translating the vast quantity of data collected with iEEG into neuroscience discoveries is difficult. RAVE eases discovery by providing a powerful, comprehensive, free, user-friendly toolkit that makes it easy to analyze and view iEEG data. For most existing solutions, users interact with their data by coding, often in Matlab. RAVE eliminates the necessity for programming expertise. Simply clicking on an electrode prompts immediate display of a number of useful analyses including the spectrogram, the response amplitude over time, the response to each individual trial, and the time series of the response at every trial. In contrast to current solutions, modifying any analysis parameter in the RAVE GUI (such as the time-frequency analysis window) instantly updates all analyses and visualizations. Development of RAVE is guided by five design principles.

### 4.1. Rigorous statistical methodology

The linear mixed-effects model used in the *Group Analysis* module incorporates our current understanding of the most rigorous way to analyze iEEG data. Individual electrodes and subjects are modeled as hierarchical random effects. Many iEEG analyses treat electrodes and subjects as fixed effects, creating the illusion of immense statistical power since there are thousands of individual experimental trials. However, the high variability across electrodes and subjects makes this analysis a poor fit to iEEG data.

No software with a useful degree of flexibility can prevent users from making statistical errors such as circular or biased analyses. However, by making the analysis transparent and easy to reproduce, other investigators may easily examine claims based on iEEG data and determine if the approach is sound. Furthermore, for users who rely on the RAVE GUI, all of the code is already shared. This permits community-based examination of the internal workings of RAVE and for any errors that are discovered to be corrected. This is in sharp contrast to current practice, where each trainee in a single laboratory might analyze data using a different combination of functions called in different order with different parameters. This can lead to inconsistency if not outright errors and make it very difficult to reproduce analyses.

### 4.2. Keep users close to the data

The *Power Explorer* module is designed for interactive data visualization to power new discoveries. Data is automatically displayed broken down by individual trials, sorted by conditions. This is important because many iEEG discoveries (as in the rest of science) are serendipitous. For instance, in an experiment originally designed to examine differences between auditory and audiovisual words, RAVE users noticed differences between different stimulus exemplars explained by the relative timing of the auditory and visual speech (Karas et al., 2019). Experimental conditions can be defined on-the-fly, with the results instantly viewable. This is more flexible than other workflows (such as generalized linear model specification in analysis of BOLD fMRI data) in which the trials making up each condition, and the contrasts between the conditions must be prespecified.

### 4.3. Run anywhere

RAVE has been successfully used on Mac, Windows PCs, Linux boxes and iPads. The use of a web browser for user interfacing means that RAVE development is “future proofed” against obsolescence of specific graphics libraries, operating systems, and processing architectures.

As biomedicine moves towards funding-agency and journal enforced data archiving and sharing, the ability of RAVE to operate in the cloud will become increasingly important. RAVE automatically organizes all data and meta-data for each participant into a project directory; the entire project directory can then be uploaded to a data archive as mandated by many journals and funding agencies. The organized directory structure and analysis files created by RAVE are also easily translatable into nascent formats such as Neurodata Without Borders (Teeters et al., 2015) and the Brain Imaging Data Structure for iEEG (Holdgraf et al., 2019).

### 4.4. Reliability and reproducibility

Replicating iEEG analyses can be challenging. Even if the code used for an analysis is available, changes in hardware, operating systems, and dependencies between different software tools can make it impossible to load data or execute the analysis code. A good solution to this problem is the use of the *Docker* system. A single binary image is shared that contains all of necessary software, data, and metadata necessary for replication (Boettiger, 2015). The RAVE website maintains a current Docker image with a complete RAVE install and demo data.

### 4.5. Play well with others

There are a number of outstanding EEG and MEG tools, including EEGLAB (Delorme and Makeig, 2004), FieldTrip (Oostenveld et al., 2011) and MNE (Gramfort et al., 2013; Gramfort et al., 2014). The RAVE website (https://openwetware.org/wiki/RAVE:SoftwareToolsTable) maintains a comprehensive list of iEEG software tools that includes the development language for each tool. RAVE is developed using “R”, a free, open source statistical language with a rich framework of existing packages developed by leading statistical and machine learning researchers (Computing, 2013). One disadvantage of “R” is that it is not as popular for scientific programming as Python, Matlab, or C and its variants C ++ and C#. For users of other programming languages or packages, RAVE is designed to integrate easily with existing tools via data exchange through open file formats or direct function calls via API for access to internal data structures. DICOM and NIfTI formats are supported via oro (Whitcher et al., 2011) and JSON via jsonlite (Ooms, 2014). Interactions with C ++ are supported with Rcpp (Eddelbuettel, 2013); reticulate supports Python; and R.matlab supports Matlab. The RAVE API permits RAVE users to adapt existing analysis modules or create new analysis modules in a variety of languages. For instance, a module to perform time-frequency analysis using Hilbert transforms could be implemented in “R”; time-frequency analyses conducted in the Matlab toolbox chronux (http://chronux.org/) could be accessed via R.matlab and displayed with the RAVE viewer.

RAVE imports and exports data in a variety of formats, simplifying the exchange between software platforms. For example, there are a number of processing pipelines for localizing iEEG electrodes such as iELVis (Groppe et al., 2017) and img_pipe (Hamilton et al., 2017), that generate CSV files that RAVE directly reads for use in the surface/volume viewer. On the export side, *Power Explorer* generates FST files so that initial preprocessing and analyses can be performed in RAVE before exporting the data for more complex analyses not currently implemented in RAVE, such as deep learning.

### 4.6. Future directions

Future development of RAVE will focus on a number of areas, guided by user demand. The existing surface and volume viewer can display cortical surface models, CT, and volumetric MRI. The ability to display diffusion MRI or blood-oxygen level dependent functional MRI (BOLD fMRI) data would be an obvious extension. RAVE supports a variety of data quality checks and referencing schemes. Individual electrodes can be excluded from analyses at any stage, and outlier/artifact trials can be detected visually in the Power Explorer GUI and removed by clicking on the outliers in a scatter plot. Future development could include additional preprocessing routines for artifact rejection or noise reduction, such as independent components analysis.
